# Supplementary material for: Are carbon emissions trading and green financial instruments synergistic? -Comprehensive quantitative research based on content analysis
Source: PLoS One. 2024 Mar 7;19(3):e0298601. doi: 10.1371/journal.pone.0298601 (PMC10919601; doi:10.1371/journal.pone.0298601)
Supplement: S1 Data — (ZIP) [file pone.0298601.s001.zip › Data/地级市Z_F工作报告.zip!/╝¬┴╓/┴╔╘┤╩╨/└┤╫╘╣1⁄2╓┌║┼í╛┬φ┐╦╩2╛▌═°í┐_2018.docx]

各位代表：

　　现在，我代表市政府向大会报告工作，请予审议，并请市政协各位委员提出意见。

　　一、2017年工作回顾

　　过去一年，我市发展面临多重困难和严峻挑战。在市委的坚强领导下，全市人民紧密团结在以习近平同志为核心的党中央周围，以迎接党的十九大胜利召开和学习贯彻党的十九大精神为主线，坚持稳中求进工作总基调，努力践行新发展理念，攻坚克难，砥砺前行，千方百计稳增长，大力推进经济结构调整，夯实发展基础，增强发展活力，提升发展质量，人民生活进一步改善，社会保持和谐稳定，全市创新转型发展呈现新局面。

　　经济增长步伐稳健。保持有效投资力度，发挥内需和外贸双拉动作用，经济运行处在合理区间。建设5000万元以上项目142个，其中新开工建设50个，完成投资96亿元，开复工率100%。吉诺高性能树脂、鸿图锂电隔膜二期、青岛昌盛光伏农业一期等23个项目竣工投产。云动力智能机器人元器件、百康药业二期、同城医药物流中心、盛吉美模块化厨房等项目加快推进。效益指标和先行指标提质趋好。在消化以往一次性收入和非税收入较高基数的基础上，预计地方级财政收入实现18.3亿元，税收收入占比提高12.1个百分点。多方筹资保证民生等方面需要，财政支出增长11%。居民消费价格指数100.7，物价总体水平稳定。城镇居民登记失业率控制在3%以内。全社会用电量增长25%，其中工业用电量增长35%。全市33大类工业行业中，近八成产值同比增长，亏损企业数量下降15%，规上工业利润总额增长34%。民营经济持续活跃，民营工业产值占全市工业92.5%，民间投资占全市投资85.8%，均列全省第一。开展“千企促销”行动和“促消费、稳增长”百日攻坚收到实效，预计社会消费品零售总额增长8%。铝型材、袜制品国际市场占有率不断提高，外贸出口增长22%。我市获批国家级出口袜制品质量安全示范区，实现吉林省工业制品出口示范区零的突破。稳步推进供给侧结构性改革，完成煤炭去产能任务，妥善处置“僵尸企业”。商品房待售面积下降21%。减免取消行政事业性收费和政府性基金13项，发放就业贷款8637万元，助保金池为企业融资1.34亿元，规上企业资产负债率同比下降3个百分点，主营业务成本低于全省平均水平。我市被省政府评为贯彻落实国家和省重大政策措施成效显著市。

　　产业结构不断优化。预计全市规上工业总产值、增加值分别增长6.3%和3.5%，两大主导、五个特色优势产业产值增长7.5%，其中新能源产业产值增长25%。推动工业转型升级。实施重点技改项目246个，辽矿集团、鑫锐工矿等企业启动转型，新进规上工业企业30户。利源精制、富奥泵业等企业产值增长25%以上。鸿图锂电隔膜与金冠电气实现并购重组，博大制药和唐豆豆食品挂牌新三板。加快农业结构调整。新建高标准农田20万亩，在调减籽粒玉米48万亩情况下，粮食产量达到47.7亿斤新高。农村土地流转95万亩，新增设施农业8629亩，榛子、蓝莓等林果经济快速发展，食用菌生产规模达到5000万袋，畜禽规模化养殖比重达到80%以上。

　　省级以上农业产业化龙头企业达到24户，市级以上115户，金翼蛋品鲜蛋加工能力保持世界第一。中德辽河源现代农业生态示范区一期工程成效显著，二期项目启动建设，现代农业产业园区发展到210个。辽源大米进入吉林大米产业联盟。我市获得中国绿色生态农业特别贡献奖。东辽县在全省县域经济综合考核中跃升9位。扩大服务业发展规模。实施服务业重点项目28个，新增服务业主体3579个，规上服务业营业收入增长8%，旅游总收入增长25%，电商交易额达到12亿元，服务业对经济增长贡献率达到37%，同比提高4个百分点。提高科技创新能力。构建与中科院长春“三所”、吉林大学等单位的产学研协同机制，争取国家和省级科技创新项目36个，科技孵化器在孵企业397家，国家高新技术企业达到9户。建立省级企业技术中心26个，15户企业被认定为吉林省第一批“科技小巨人”，占全省比重超过10%。创建市级科技示范园区和科普示范基地40个，有效专利达到645件。经济开发区创建省级高新技术产业开发区通过专家评审。

　　改革开放富有成效。重点领域改革向纵深推进。行政审批事项进厅率达到99.7%，审批环节减少11.8%，“一次性跑动”占比50.4%，平均办事时间压缩85%以上。我市营商环境在国务院第四次大督查指标抽样调查中取得良好成绩。政务服务“一张网”建设实现突破，市政府门户网站跻身全国地市级20强。投融资平台打造和渠道拓展取得重要成果，辽源城投集团、吉林金控集团辽源市农村金融综合服务中心、西安榆银村镇银行投入运营，设立全市首支产业转型升级基金，东丰农村商业银行完成改制。安全生产领域改革不断深化。国有林场、农村土地“三权分置”改革进展顺利。不动产登记、城市综合执法和公务用车改革基本完成。研究启动经济开发区体制机制改革。辽矿集团列入国家第二批增量配电业务改革试点。不断扩大对外交流合作。组织参加东北亚博览会等经贸交流展会，赴北京、上海、广东、浙江、福建等地开展务实精准招商，积极对接中国核电、广药、神华、华为等知名企业，洽谈项目269个，签订战略协议26个，签约项目107个，德兴探感生态养殖、蓝天城儿童欢乐世界等项目落地开工。启动与绍兴对口合作，上海漕河泾开发区在我市设立产业转移促进中心，成立北京吉林辽源商会，同域外商协会的联系交往日益强化。

　　城乡建设扎实有序。伊开高速二期、集双高速辽源段开工建设，完成辽西线安全生命防护工程和辽白一级路维修改造。新改建农村公路400公里。辽源至北京直通列车、辽源至长春客运城际列车开通运行，四通高速铁路前期和辽源机场选址工作进展顺利。“气化辽源”、“引松入辽”等重大工程加快实施。以城市规划建设管理提挡升级攻坚行动为抓手，改造建设市政街路34条，建设各类管网67公里，9个海绵城市建设工程同步开工。创建城管示范街3条、严管街8条，城区机械化清扫率达到60%。优化市区公交线路30条，更新新能源公交车36台、出租车330台，施划机动车停车泊位1.3万个，投放公共自行车400辆。治理和修复采煤沉陷区废弃地28公顷，困扰多年的矿山治理项目设计变更获省批复。职教园区8.2公里地下管廊投入使用。东北卓越云计算中心安全策略全面升级，大数据交互共享平台在全省率先完成。时空大数据与云平台项目列为国家测绘地理信息局试点。建成市级公共资源电子交易平台。金州、那丹伯、寿山等重点镇基础设施建设逐步完善，公共服务能力不断提升。实施农村减排、连片整治项目160个，改造农村危房353户，解决农村饮水安全问题9015人，完成农村厕所改造5200户。建设省级美丽乡村4个。东丰县成功创建国家级卫生县城。

　　民生福祉持续增进。以增加公共财政投入为依托，多措并举，如期完成年度71件惠民实事。把脱贫攻坚作为重点任务，精准推动产业扶贫，精心组织教育和医疗扶贫，年度实现减贫5753人，退出贫困村16个，“五点双享两救助”脱贫保障机制被评为2017年全国脱贫攻坚奖创新奖。城镇新增就业4.7万人、扶持创业6897人，转移农村劳动力18.7万人次。预计城乡常住居民人均可支配收入分别增长4.9%和5%。养老金、失业金和城乡低保同步提标，社会救助受益群众达到4.6万人。顺利通过全域国家义务教育均衡发展复检。全面启动城区义务教育阶段学生营养配餐计划。职教园区一期主体工程竣工，东北袜业纺织工业园技师学院挂牌成立。开展公立医院改革，取消药品加成、实行零差价，人均住院费用下降7.14%，首批接入国家跨省异地就医结算平台。安置保障性住房4520套。城区新增居民管道天然气用户2万户。农网光纤入户覆盖28个乡（镇）、3万余户。送戏下乡、广场汇演等群众文化生活和吉剧创作等文化事业加快发展。侵华日军辽源高级战俘营旧址展览馆被确定为全国爱国主义教育基地。建设全民健身中心等体育设施，全省民族运动会我市获奖牌数量取得历史性突破。“河长制”工作全面铺开，仙人河黑臭水体治理工程启动实施。淘汰燃煤小锅炉186台、黄标车2606台。林地清收还林2.45万亩，完成省计划182%。强力解决中央环保督察群众反映问题292件，严肃查处了一批环境违法案件。

　　社会大局和谐稳定。健全完善立体化社会治安防控体系，创新实施“袜乡管家”工程，全市刑事、治安案件分别下降14.5%和5.4%，党的十九大安保维稳工作实现“两个绝对平稳”、“三个绝对安全”。我市连续两年被评为全国社会治安综合治理优秀城市。“七五”普法实现良好开局，宪法主题公园和法治走廊如期建成。及时回应群众诉求，依法维护群众合法权益，一批信访突出问题得到妥善解决。12345市民服务热线正在成为资源整合集约、贴近融入百姓和服务规范高效的新平台。深入开展“安全生产责任深化年”和“五个一工程”建设活动，各类生产安全事故下降7.5%。食药监管“三个中心”项目投入使用。连续33年实现无重大森林火灾。保持民族团结、宗教和顺良好局面。积极推进国防教育和双拥共建。广播电视、外事侨务、方志、档案、气象、地震、人防、供销社等工作和妇女儿童、老龄、残疾人、关心下一代等各项事业全面发展。

　　政府建设得到加强。落实全面从严治党要求，推进“两学一做”学习教育常态化制度化。加强法治政府建设，坚持科学民主决策，健全政府党组工作规则，制定自觉接受市人大法律监督、工作监督和市政协民主监督的实施意见。提请市人大常委会审议煤矿文化遗产保护条例，接受水源地保护专题询问，配合市政协开展医药健康产业发展、老年服务体系建设等调研视察，办理代表建议和委员提案249件。公开预决算、“三公”经费等政府信息1.8万余条，政府采购节支率5.2%。开展专题督导检查56次，加大跟踪问效力度，推动工作落实。严肃查处违反中央八项规定精神、“四风”和侵害群众利益问题，惩防腐败体系不断强化。

　　各位代表，回顾过去一年，我们面对宏观经济下行和结构调整的阵痛，面对国家和省一系列督察、检查、审计、巡视发现问题整改的要求，面对从严整治生态环保突出问题的压力，面对政府性融资、债务监管和经济运行监测、统计监督等政策的变化，面对增收难度加大与政府偿债高峰期到来的矛盾，面对维护安全稳定的艰巨任务，这些要求新、标准高、难度大的考验集中在一起，使得各种矛盾叠加、困难隐患交汇的复杂局面多年未有，需要咬紧牙关、攻克难关的艰辛努力多年未有。在这种情况下，各项工作的成效和进展实属不易，经济社会发展呈现诸多向好变化尤为可贵。这是省委、省政府和市委正确领导、科学决策的结果，是市人大、市政协和社会各界有效监督、鼎力支持的结果，是各县（区）、各部门和全市人民团结拼搏、极大付出的结果。我代表市政府，向奋斗在各条战线上的广大干部群众，向离退休老领导、老同志，向各民主党派、工商联和人民团体，向人民解放军驻辽源部队指战员、武警官兵和公安干警，向中省直各单位以及所有关心支持辽源发展的各界朋友致以衷心感谢和崇高敬意！

　　我们也清醒看到，我市经济总量小、底子薄，应对经济下行和市场风险的韧性弱、回旋余地小，产业增量提质和结构优化升级的任务艰巨繁重；资金、征拆、要素成本等方面制约还没有得到很好破解，实体经济发展和一些企业生产经营困难较多，重大项目储备不足，后续投资增长乏力；政府债务负担重，财政持续增收难，刚性支出增多，收支矛盾不断加剧，财政风险隐患不容忽视；基础设施建设和公共资源供给与城乡居民期盼还有较大差距；绿色发展和社会安全稳定的根基仍不牢固；部分政府公职人员的思想作风、工作能力、服务水平与新时代新要求不相适应。我们一定增强忧患意识和担当精神，采取有效措施解决这些问题，尽心竭力做好政府工作，决不辜负全市人民的重托。

　　二、2018年工作安排

　　今年，是贯彻党的十九大精神的开局之年，是改革开放40周年，是决胜全面建成小康社会、实施“十三五”规划承上启下的关键一年。做好市政府工作，必须以习近平新时代中国特色社会主义思想为指导，全面贯彻党的十九大精神，认真落实省委、省政府和市委决策部署，坚持稳中求进工作总基调，坚持新发展理念，按照高质量发展的要求，以供给侧结构性改革为主线，统筹推进稳增长、促改革、调结构、惠民生、防风险各项工作，加快推动发展质量变革、效率变革、动力变革，在打好防范化解重大风险、精准脱贫、污染防治攻坚战方面取得扎实进展，保持经济社会持续健康发展，努力谱写辽源创新转型发展新篇章。

　　发展的主要预期目标是：地区生产总值增长5%左右，居民消费价格涨幅在3%以内，城镇新增就业3.3万人以上，登记失业率控制在3.9%以内，城乡居民收入与经济增长基本同步，节能降耗完成省计划。

　　综合分析各方面情况，今年我市发展仍将面临错综复杂的外部环境，自身补短板、强弱项的效果也绝非一时可得，必须继续做好打攻坚战和啃硬骨头的充分准备。虽然困难和挑战会更多更大更严峻，但我们迎难而上的意志坚定不移，克难制胜的信心不可动摇。作为老工业基地和资源枯竭转型城市，这些年辽源的振兴发展和转型升级就是在爬坡过坎、滚石上山中走过来的。党的十九大对推进新时代中国特色社会主义事业作出了全面安排部署，全省走出振兴发展新路有国家政策支持、清晰路径举措和坚实工作基础，正处于重要战略机遇期，我市已初步形成接续替代产业体系，目前经济发展要素运行平稳，发展态势稳中有进，同时拥有良好政治生态和社会氛围。只要保持战略定力、守住各条底线，万众一心，共克时艰，一步一个脚印向前迈进，辽源转型发展就一定能够闯关夺隘、化蛹成蝶，不断创造新的辉煌。

　　今年，要重点抓好以下几个方面工作：

　　（一）着力提高经济发展质量。围绕建设现代化经济体系战略目标，坚持质量第一、效益优先，深化供给侧结构性改革，提高全要素生产率，切实推动高质量发展。大力推进项目建设。盯紧国家政策导向和市场发展需求，突出新一轮东北振兴战略支持、发达地区产业转移、域内企业转型升级、靠大联强资产重组，集中力量上一批大项目好项目。实施投资5000万元以上项目127个，重点推动高精铝加工、新能源新材料、医药健康骨干企业项目建设，深入谋划实施生态环保、民生和基础设施项目，滚动保持重大项目储备200个。厚植民营经济优势。实施中小微企业培育工程，新建10个企业孵化器和创业基地，培养10户以上“小升规”企业，生成中小微企业400户左右。民营经济主营业务收入、上缴税金分别增长6%和3%。开展“温商暖企”行动，强化贴心精准服务，落实减费降税政策，推动金融服务和产品创新，缓解融资难、融资贵，帮助企业降本增效。激发和弘扬企业家精神，维护企业合法权益，清理废除妨碍统一市场和公平竞争的各种做法，让投资者安心创业、放手发展。积极拉动消费和出口增长。扩大个性化消费供给，积极推动汽贸服务一体化集聚区建设，辐射带动周边区域消费。完善商品住房销售促进措施，活跃二手房市场，建立多主体供给、多渠道保障、租购并举的住房制度。继续加强3个出口基地建设，支持引导企业开展跨境电子商务，让辽源装备、辽源棉袜、辽源绿色农产品更多地走出国门、走向世界。优化存量资源配置。继续落实煤炭去产能目标任务，完善“僵尸企业”处置制度，采取一企一策办法，引进战略投资者盘活存量。开展全市土地资源利用专项整治，提高空置土地和低效工业用地利用效率。进一步提高保障房货币化安置比例，促进商品住房库存资产转化。再清理一批涉企收费项目，落实阶段性失业保险费率政策，有效降低实体经济制度性交易成本。严防财政和金融风险。按照量入为出、量力而行原则，健全完善“借、用、还”政府融资管理机制，建立债务管理负面清单制度，实施市级财政零基预算，加强出借政府性资金清收，严格控制政府债务增量。坚决清理整顿互联网和交易场所违法违规业务，有效防范打击非法集资行为，守住不发生系统性区域性金融风险的底线。建立健全征信体系，加强信用信息共享，构建“一处违法、处处受限”的联合惩戒机制。

　　（二）着力以创新引领结构优化升级。坚持以科技和人力人才资源为支撑，以创新推动新旧动能转换，改造提升传统产业，培育壮大新兴产业，提高创新驱动发展能力。加快工业经济转型升级。深入推进产业转型升级行动计划，滚动推进重点工业转型项目92个。引导利源精制、麦达斯铝业等企业提升智能化制造层级。支持鸿图锂电隔膜、中聚新能源等企业扩大规模，对接我省新能源汽车产业联盟，力争配套基地布局辽源。推动吉林装备、聚源重型等传统制造企业向智能机械设备制造方向发展。扶持瑞意粮机、长江胶管等“隐形冠军”企业扩能升级。务实推进与华为公司合作的云计算大数据产业项目。鼓励建筑房地产企业向制造业和网络信息等新兴业态转型。持续推动服务业攻坚。重点加强以兴辽物流配送为依托的物流业主导区建设，以东北袜业为依托的特色产业小镇建设，以中小企业服务大厦为依托的创业服务区建设，以龙山科技智慧产业园为依托的信息产业区建设，带动关联企业集约化发展。建立旅游资源联动、线路联接、产品联合发展模式，完善旅游景区基础设施建设，打造全域休闲旅游品牌。加快农村电商普及推广，鼓励村集体、合作社、新型职业农民领办电子商务，全年发展电商网店1000个，村级电子商务服务站覆盖率达到60%以上。促进产学研深度融合。深化政府、企业同科研院校合作，依托我市省级针纺、化药、蛋品中试中心，推进一批重大科技攻关、成果转化和创新项目。完善科技大市场服务功能，加快培育创新型企业和高新技术企业。新建院士工作站1个，省级科技创新中心1个，实施省级科技成果转化项目20个以上。培育发展“科技小巨人”企业10户以上。大力实施品牌和人才强市战略。壮大“辽源制造”，鼓励“辽源创造”，严格行业生产标准质量体系，加强知识产权保护。推动域内同质企业走产业联盟发展模式，开展增品种、提品质、创品牌专项行动，提升辽源品牌的竞争力和影响力。认真落实“152”人才集聚工程，建立本地学子才子回引计划和柔性引才机制，力争引进一批高层次的管理型、技能型和基础型人才。完善产教融合模式，依托职教园区订单定向培养实用型“高级蓝领”。创立省级首席技师工作室、师徒工作间，大力培养推荐省级拔尖创新和国家级“百千万”人才。

　　（三）着力实施乡村振兴战略。按照产业兴旺、生态宜居、乡风文明、治理有效、生活富裕总要求，科学制定乡村振兴战略规划，建设精品农业市和畜牧业强市，加快实现农业农村现代化。推动城乡融合发展。坚持以工补农、以城带乡，加强金州省级示范镇和那丹伯、横道河、渭津、安恕、辽河源等节点镇基础设施建设，健全基本公共服务体系。推进农业农村“放管服”改革，破除一切束缚农民手脚的不合理限制和歧视。推动户籍制度改革和居住证制度双落地，加快农村土地确权登记颁证，研究确定“三权分置”有效办法，探索农村土地征收、集体经营性用地和农民宅基地入市措施。鼓励引导工商资本参与农村振兴，建立农村金融服务机构，设立运营农业发展基金。推进农业供给侧结构性改革。坚持质量兴农、绿色兴农，健全完善现代农业产业体系、生产体系、经营体系。科学调控籽粒玉米种植面积，新建高标准农田13万亩，稳定粮食综合生产能力。推广优质经济作物种植，推进特色农畜产品标准化规模化生产，新建棚室8500亩。加强黑土地保护，推广化肥农药零增长和测土配方施肥等技术。加快农产品检验检测基地建设，建立农产品可追溯体系。发展市级现代农业园区10个以上，培育“三品一标”4个以上，支持东丰、东辽创建国家农产品质量安全县和有机产品认证区。健全农村一二三产融合体系。稳步提升土地流转面积，有序推进土地适度规模经营，培育发展家庭农场、农民合作社及联合社等新型经营主体。依托产业化龙头企业，推广“互联网+”精品营销模式，构建集金融、保险、防疫、流通、信息于一体的现代农业服务业，促进小农户和现代农业发展有机衔接。支持有条件的乡村建设循环农业、创意农业、农事体验田园综合体。加强美丽宜居乡村建设。持续改善农村人居环境，开展突出环境问题综合整治，建立市场化多元化生态补偿机制，实现百姓富、生态美的统一。推进“厕所革命”，改造农村厕所2万户。新创建省级美丽乡村4个、省级特色小城镇试点11个。

　　（四）着力深化改革扩大开放。坚定不移创新体制机制，不断提高开放型经济水平，为经济发展注入新动力、增添新活力、拓展新空间。推动重要领域和关键环节改革。深化“放管服”改革，推行“一窗受理、集成服务”，进一步提高行政审批和公共服务效率。深化商事制度改革，全面推进“双随机、一公开”监管。加强政务服务“一张网”建设，积极打造24小时网上政府和“移动的门户”，让企业和群众办事更方便、高效、舒心。严格涉企收费目录清单管理，防止不合理收费死灰复燃。加强政银企合作，组建市县两级物权公司，建立优质企业持续融资机制。尽快做大做优城投集团业务，提升投融资、服务实体经济和经营城市能力。支持东北袜业、金翼蛋品等9户企业列入全省上市培育计划。深化国有企业改革，继续推动辽矿集团剥离“三供一业”。扎实推进开发区体制机制改革创新。务实推动区域合作和招商引资。加强与浙江绍兴对口合作，推动袍江经济开发区在我市建设“飞地”经济园区，支持东北袜业与诸暨深度合作。积极承接上海漕河泾等地产业转移。按照全省对外开放总体布局，加强与四平、铁岭、通辽互动交流，推动四市经济协作区建设。深化与德国在生态农业、职业教育等领域合作。充分借助国际国内高层平台、构建自主招商平台、打造专题对接平台，开展精准招商引资。注重人才招商、资源招商、服务招商，建设高质量项目库，发挥本地企业家、域外辽源籍人士作用，实现多渠道宽领域招商，力争全年落地开工项目20个以上。

　　（五）着力提升城市功能和品位。科学修编城市总体规划，不断完善功能、丰富内涵。抓好基础设施工程。伊开高速二期建成通车，续建集双高速辽源段，积极推动四通高速铁路、东南环城高速公路项目列入国家和省路网规划及开工计划。完成辽源机场选址。加强“村村通”公路养护，新建农村公路100公里。推进“引松入辽”干线建设和辽东支线前期工作，继续实施东辽河城防工程。扩大“气化辽源”覆盖区域。抓好市政提标工程。推动福镇大路、财富北路等城区道路改造，加快解决“断头路”问题。继续实施城市地下综合管廊工程和海绵城市建设。改建供排水、供热管网52公里。推进寿山净水厂和市污水处理厂建设。争创国家级停车场建设试点，市区规划新建一批立体停车场，探索城区重点区域停车收费。新建改造一批城区公共厕所。抓好管理服务工程。拓展智慧城市服务功能，完善网站、手机、电视及终端机一体化网络，市区实现智慧社区建设全覆盖。实施城市管理“721”工作法，深入开展市容环境“四乱”专项治理，持续推进“禁烧”、“拆违”攻坚。建立“零工劳务市场”，迁移宠物市场。深化动静态交通秩序整治，缓解城区重点路段交通高峰期拥堵问题。抓好绿美人文工程。实施城市重点区域美化提升和夜景观艺术照明工程。新建改造城市景观带5个、街头绿地20处，完成林苑公园二期建设。探索推广环卫保洁市场化运作模式，城区机械化清扫率达到70%以上。凝炼城市精神，倡导文明礼让、见义勇为、扶危济困等良好社会风尚，不断提升市民文明素质，让辽源成为有魅力、有温度、有情义的秀美和谐之城。

　　（六）着力加强生态文明建设。牢固树立“绿水青山就是金山银山”的理念，加快实施项目工程，健全完善监测体系，抓紧抓实中央环保督察反馈问题整改，推动人与自然和谐共生、协调发展。坚决打好污染防治攻坚战。全面深化“河长制”，以仙人河黑臭水体治理和饮用水水源地保护为重点，集中开展清理河道垃圾、取缔沿河两岸污水直排、治理畜禽养殖污染和打击河道采砂等专项行动，有效改善境内水质。集中开展燃煤锅炉、油烟扬尘、机动车尾气、秸秆焚烧等专项整治，推进新型能源替代工程，力争全年空气优良天数占比达到80%。推行农业“三减”行动计划，开展封山育林、林地清收还林，加强国家矿山湿地公园、仙人河老工业区等废弃污染土地恢复治理，推进采煤沉陷区、采矿废弃地修复和再利用。争创国家级生态修复、城市修补试点。推进绿色循环经济发展。建立支持循环经济发展的激励和约束机制，鼓励引导园区和企业实施节能改造、建立循环产业链。加快秸秆“五化”综合利用。倡导节约低碳健康生活方式，扩大生活垃圾分类收集处理试点，有序发展公共自行车等共享经济，推动资源节约型城市建设。构建生态环保长效机制，健全工作责任体系，建立自然资源资产负债表和环境准入清单。完善环境风险评估、隐患排查、事故预警和应急处置工作机制，强化综合执法监管，坚决制止和严厉惩处破坏生态环境行为。

　　（七）着力推动以保障改善民生为重点的社会建设。竭尽全力办好民生实事，切实增强群众的获得感、幸福感、安全感，重点实施“一确保、五提升、一健全”：确保打好打赢脱贫攻坚战。巩固市县乡村四级联动机制，注重务实精准扶贫，注重扶贫同扶志扶智相结合，突出产业支撑、项目带动，建立推广贫困群众入股分红等工资性收入机制。整合政策资源打好“组合拳”，用足用好“五点双享两救助”政策。争创全省金融信贷扶贫先行试点。年度实现稳定脱贫4000人，脱贫贫困村18个。提升教育发展水平。扩大普惠性学前教育资源，建立名校集团化和大学区管理制度，实施“薄弱校”改造工程，推动城乡义务教育一体化发展。启动高考改革准备工作。加强师德师风建设，培养高素质教师队伍。职教园区一期投入运行，启动二期建设工程，努力打造全省领先、全国有名的职教品牌。提升社会保障能力。完善职业培训、就业援助和就业创业平台，突出困难就业群体，失业人员再就业6000人，转移农村劳动力15万人次。完成养老与失业金调待、城乡低保提标，实现农村低保与扶贫标准“两线合一”。健全治理农民工欠薪工作协调机制，调整机关事业单位、企业退休人员养老金和居民基本养老保险待遇。健全完善社会救助体系。提升住房保障质量。加快推进棚改工作，新建和货币化安置保障房1300户，综合整治老旧住宅小区10个。推动实施城乡结合部、城区危旧楼房维修改造。下大气力解决小区物业弃管问题，推广物业企业与业主双向约束机制经验。改造农村危房370户。启动电梯安全工程。提升全民健康服务。深化公立医院综合改革，促进优质医疗资源向基层延伸，构建30分钟基层医疗服务圈。持续推进“十百千”城乡居民健康管理工程，全面开展“家庭医生”签约服务，“特殊群体”签约率实现100%。免费开展全市贫困人口白内障救治。鼓励支持社会资本进入医疗卫生领域。围绕推进医养结合，建立完善养老、孝老、敬老、惠老的服务体系、政策措施和社会环境，加快老龄事业和产业发展。提升文化生活品质。创建全国第四批公共文化服务体系示范区，进一步提升基层文化活动室的使用功能。建成并开放一批文体场馆。举办首届市民健身运动会。办好“辽源杯”琵琶大赛。组织开展吉剧振兴作品展演，鼓励创作更多集思想性艺术性于一体的精品力作。健全公共安全体系。深化平安辽源建设，完善防控运行机制，大力整治社会治安突出问题，维护国家安全和社会稳定。深入实施“七五”普法规划，建设公共法律服务体系，基本实现村级法律顾问全覆盖。加强文化市场监管，净化网络环境。健全完善社会矛盾预警化解机制、利益诉求表达机制和救济救助机制，认真解决群众合理诉求。强化安全生产“六大体系”建设，坚决遏制重特大事故发生。深入实施食品药品安全战略，让人民群众饮食用药更加安全放心。加强全民国防教育、双拥共建，推进军民融合发展。

　　各位代表，面对新时代提出的新任务、新要求，市政府一定把发展和民生牢记在心，进一步强化法治思维，依照宪法法律行使职权、履行职责、开展工作，自觉接受人大、政协和社会各界监督。大兴调查研究之风，弘扬敬业专业精神，更多深入到基层一线和困难突出、情况复杂的地方，敢担当、不推诿，善作为、求实效。完善各项工作的督促检查和考核评价体系，保护干事创业热情，为忠诚事业、大胆探索创新的人撑腰鼓劲。认真落实全面从严治党要求，加强党风廉政建设，保持反腐高压态势，整肃庸政懒政怠政行为，切实以良好作风、严明纪律和工作实绩回应全市人民的期待。

　　各位代表，走进新时代，共赴新征程。让我们更加紧密地团结在以习近平同志为核心的党中央周围，高举习近平新时代中国特色社会主义思想伟大旗帜，在市委的坚强领导下，不忘初心、牢记使命，勇于担当、真抓实干，奋力推进辽源创新转型发展，为全市人民生活得更加幸福美好而不懈奋斗！
